# Prevalence of Depressive, Anxiety, and Stress Symptoms Among Dentists at an Academic Institution: A Cross-Sectional Study

**DOI:** 10.3390/healthcare14182908

**Published:** 2026-09-08

**Authors:** Sumaya O. Basudan

**Affiliations:** Department of Restorative Dental Sciences, College of Dentistry, King Saud University, P.O. Box 60169, Riyadh 11545, Saudi Arabia; sbasoudan@ksu.edu.sa

**Keywords:** DASS-21, dentists, mental health, occupational stress, academic faculty, Saudi Arabia

## Abstract

**Background**: This study aimed to investigate the prevalence and levels of depressive, anxiety, and stress symptoms among dentists at an academic institution, and to identify demographic and work-related factors correlated with these psychological conditions. **Methods**: A cross-sectional study was conducted among full-time dentists teaching at King Saud University (Riyadh, Saudi Arabia). Data were collected using a self-administered questionnaire that captured demographic and work-related variables, alongside the validated 21-item Depression, Anxiety, and Stress Scale (DASS-21). Data were analyzed using descriptive statistics, Chi-square tests, and multiple linear regression to determine significant associations. **Results**: A total of 114 participants completed the questionnaire, of which 86.8% were faculty and the rest non-faculty. The prevalence of depressive, anxiety, and stress symptoms was 21.9%, 32.5%, and 34.2%, respectively, predominantly at moderate levels. The prevalence of at least one subscale was 44.7%, and a significant correlation was found for the co-occurrence of the three subscales (*p* < 0.001). Regression analysis indicated three correlates of stress: dissatisfaction, female sex, and choosing a career in academia were significantly associated with the stress scores (*p* < 0.05). No significant factors were correlated with the anxiety or depression subscales. While females and dissatisfied participants reported higher scores for stress, intending to work in academia as a primary career choice was significantly correlated with lower levels of stress, highlighting the potential importance of intrinsic motivation for academia. **Conclusions**: A considerable proportion of participants experience depression, anxiety, and stress symptoms, suggesting the need for targeted institutional strategies to support mental well-being, particularly among vulnerable groups.

## 1. Introduction

The dental profession involves persistent physical and psychological demands that are associated with adverse health outcomes. A recent review reported the prevalence of musculoskeletal disorders and pain among dentists to range from 68% to 100% [1]. Beyond physical strain, the environment presents multiple stressors, including high workloads, scheduling constraints, and managing anxious patients. Additional stress arises from operational challenges, like staffing, administrative burdens, job dissatisfaction, and fear of complaints, alongside occupational hazards such as radiation and infectious agents [2]. Consequently, over 70% of dentists perceive their jobs as highly stressful, with reported levels exceeding the general population [3]. Chronic occupational stress is linked to increased psychological morbidity, including elevated rates of anxiety and depressive disorders [3,4,5].

Depression involves a persistent low mood and/or loss of interest in usual activities; anxiety is characterized by excessive, hard-to-control worry accompanied by physiological arousal; and stress reflects heightened emotional and physiological tension when perceived demands exceed an individual’s coping capacity [6,7]. Although distinct, these conditions frequently co-occur, and symptoms may overlap. Long-term exposure can impair performance, decrease quality of life, and lead to early retirement [3,5,8,9]. Therefore, early identification of these conditions is critical to improving professional outcomes and well-being.

While a large body of literature exists on the stress and psychological well-being of dentists and dental students, less attention is directed toward dentists in academia, who also teach, and, therefore, may be prone to even higher levels of stress as they take on combined professional roles and become susceptible to the stressors of both dental and academic environments [10]. Recently, research has pointed to high levels of distress among university faculty [11,12], and the roles of dental faculty include teaching, research, clinical practice, and administration [13]. This multifaceted workload is a known contributor to psychological distress and burnout among faculty in other health professions [14,15]. The well-being of dentists in academia is vital for educational quality and research output, as high stress levels are major contributors to faculty attrition and the global educator shortage [16,17]. Collectively, rapid turnover could create gaps in education that impact student performance and patient outcomes.

To the best of our knowledge, few studies have assessed the well-being of dentists in academia, particularly in the Middle East, with limited data on the prevalence of depression, anxiety, and stress using standardized measures. Understanding the mental health profile of this essential workforce is critical for individual well-being and for sustaining the quality of dental education and research output. Furthermore, identifying factors associated with distress is foundational for guiding institutional interventions to mitigate psychological strain [12]. Given the lack of evidence in the Arab region, the present study is the first exploratory investigation of depression, anxiety, and stress among dentists at King Saud University, which is the oldest and largest dental school in Saudi Arabia.

### Aim

This study aimed to investigate the prevalence and levels of depression, anxiety, and stress among dentists at an academic institution, and to explore demographic and work-related factors correlated with these psychological conditions.

## 2. Materials and Methods

### 2.1. Study Design and Participants

This was a cross-sectional study conducted at the College of Dentistry of King Saud University (KSU) in Riyadh, Saudi Arabia. All full-time dentists teaching undergraduate courses were eligible to participate. This included both faculty and non-faculty members. College members are employed at either one of two tracks, the academic track, typically involved with developing and teaching the dental curriculum, as well as conducting research, or the clinical track, full-time dental practitioners treating patients at the college dental clinics. They may participate lightly in clinical teaching of undergraduate students but are not involved in the rest of the curriculum or in research; they are labelled as the “non-faculty group” in this study. Members on leave, studying, or receiving any form of psychological management (cognitive or behavioral therapy, medication, or a combination; whose responses could be confounded or altered due to the effect of therapy) were excluded. This study was designed in line with the principles of the Declaration of Helsinki. Ethical approval was obtained from the Ethics Committee of the College of Dentistry’s Research Center (No. IR0096) at KSU.

### 2.2. Sample Size Calculation and Recruitment

The required sample size was determined by calculating the demands for both primary study objectives: estimating the prevalence of psychological distress and exploring associated factors through multiple linear regression. The larger of the two calculations was adopted. For the prevalence estimate, an anticipated prevalence of 50% was utilized to yield the most conservative sample size. Using a 95% confidence level and a 6% margin of error, and adjusted for a finite population of 290 dentists, the required sample size was calculated as 99 participants. For the multiple linear regression analysis, an a priori power analysis was conducted (G*Power version 3.1) [18]. To detect a medium effect size (Cohen’s *f*^2^ = 0.15) with 80% statistical power (1 − *β* = 0.80), a 5% significance level (*α* = 0.05), and up to 7 predictor variables, a minimum of 103 participants was required. To satisfy both methodological requirements, the target sample size was set at 103 participants. An additional 20–30% were added to accommodate non-responses. To ensure representative sampling, members were stratified by specialty, then multiplied by 0.5 (as a uniform sampling fraction) to calculate the required number of participants from each specialty. Within each stratum, eligible members were randomly assigned identification numbers, and individual participants were selected via a computer-generated random sequence (SPSS Version 21.0) until the quota for each specialty was reached (N = 129). Invitation letters with a hard copy of the questionnaire were initially distributed through department secretaries, inviting the 129 out of 240 dentists to participate during the beginning of the academic year of 2015. Due to a low initial response, a follow-up electronic web-based format was designed using Google Forms and sent to institutional email addresses. Recruitment was performed by independent personnel, and the author was blinded to the process. To avoid double responses, the email contained instructions for members who had already filled in the hard copy survey to not fill in the electronic one. Participation was voluntary, confidentiality and anonymity were assured, as well as assurance that data will be reported in aggregate and not reported individually. To minimize social desirability bias and lessen confidentiality concerns regarding mental health reporting, the survey platform was configured to de-link all email-tracking mechanisms from the response data. No Internet Protocol addresses or institutional login credentials were required or collected, and unique token-based links prevented multiple submissions from the same respondent. Participants reviewed an electronic informed consent page and checked a box indicating their consent and agreement to participate. An automatic reminder was sent after four weeks, the target sample size was achieved.

### 2.3. Study Tool and Procedures

A two-part, self-administered questionnaire was developed in English. There was no need for a translation since English is the main language of education and part of clinical and administrative communications in the dental school; hence, all members have advanced levels of English language skills. The first section of the questionnaire included 11 questions related to demographic data, such as gender, marital status, rank, discipline, years of experience, whether working in academia was the first career choice, number of administrative roles, private practice, level of satisfaction with students and department members, overall satisfaction at the college, and stress-coping mechanism. The second part of the questionnaire contained the 21 items from the validated Depression, Anxiety and Stress Scale (DASS-21). The last question was multi-choice, asking the participants to choose how they cope with stress from a list of different coping methods. DASS was developed by Lovibond and Lovibond to assess the core symptoms of these three psychological conditions [7]. It demonstrates satisfactory psychometric properties and reliability [19]. DASS-21 is a short form of the original 42-item DASS questionnaire, demonstrating good-to-excellent internal consistency, adequate reliability, and construct validity [20,21]. Each of the three subscales contains 7 items scored on a Likert scale from 0 to 3, where 0 = did not apply to me, 1 = applied to me to some degree, or some of the time, 2 = applied to me to a considerable degree, or a good part of the time, 3 = applied to me very much, or most of the time. Scores are calculated by summing the scores for the relevant items, multiplying by two to compare to the original DASS-42 scale and then evaluated according to the following severity rating index:

- Depression: normal (0–9), mild (10–13), moderate (14–20), severe (21–27), extremely severe (28+).
- Anxiety: normal (0–7), mild (8–9), moderate (10–14), severe (15–19), extremely severe (20+).
- Stress: normal (0–14), mild (15–18), moderate (19–25), severe (26–33), extremely severe (34+).

### 2.4. Statistical Analysis

Statistical analyses were conducted using the Statistical Package for Social Sciences version 26.0 (IBM Corp., Armonk, NY, USA). Descriptive statistics were generated using percentages and frequencies. The internal consistency of the DASS was evaluated by Cronbach’s alpha. The univariate association between the co-occurrence of each pair of psychological conditions was examined using the Chi-squared test. Spearman’s rank-order correlation values were reported to determine the pairwise relationships among stress, anxiety, and depression levels. A multiple linear regression model was used to test whether severity of depression, anxiety and stress scores was associated with the different variables in the questionnaire. Statistical significance was set at *p* < 0.05.

## 3. Results

### 3.1. Demographic Characteristics

In total, 118 of 129 invited participants completed the questionnaire, achieving a response rate of 91%. Four participants (3.4%) disclosed receiving psychological management and were excluded. The final sample consisted of 114 participants, whose demographic characteristics are presented in Table 1. Participants were equally distributed between males and females (49.1% and 50.1%), and the majority were married (79.1%). Most participants were academic faculty, while only 13.9% were non-faculty. There were 43.9% junior faculty in the ranks of demonstrators and lecturers who hold only a bachelor’s or an additional master’s degree.

### 3.2. Prevalence of Depressive, Anxiety, and Stress Symptoms

**Figure 1:**
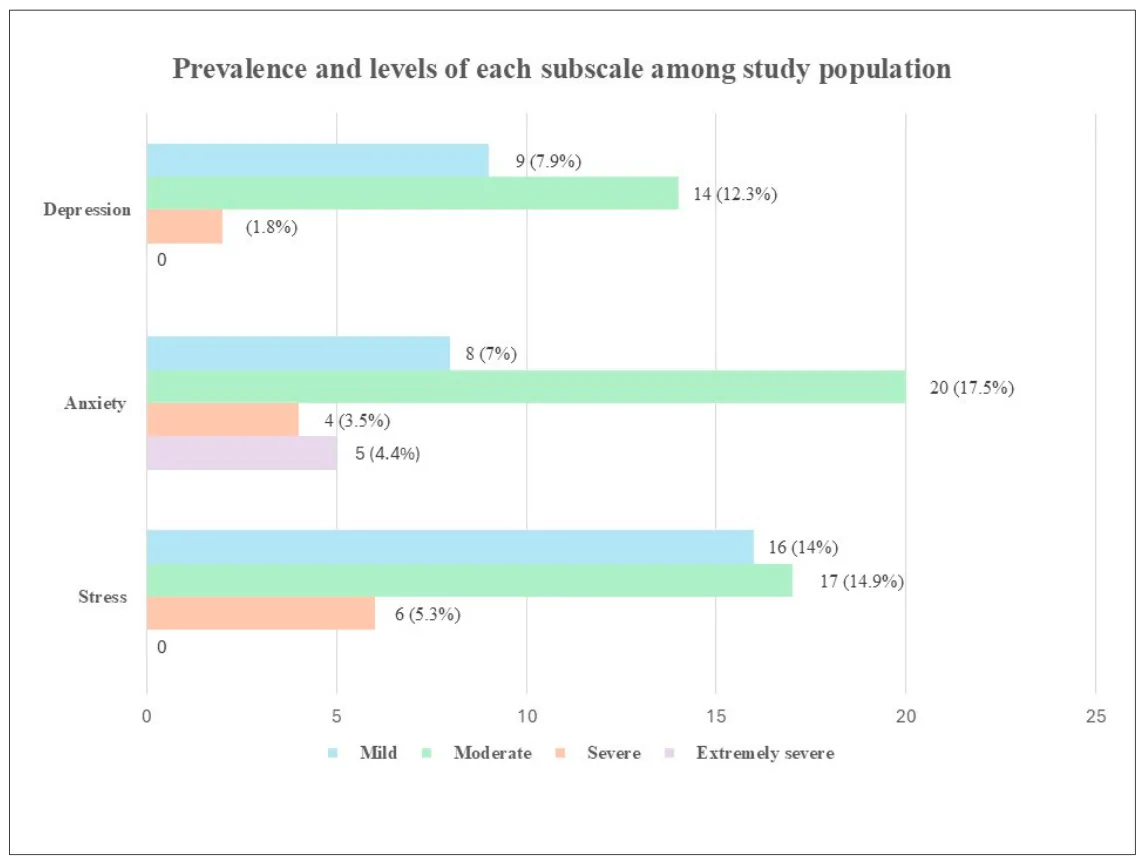
Prevalence and levels of DASS subscales among study cohort (n = 114).

Cronbach’s alphas for the subscales were calculated (depression = 0.78, anxiety = 0.78, stress = 0.86), indicating the good internal consistency and reliability of the questionnaire. The prevalence of depressive, anxiety, and stress symptoms among participants was 21.9%, 32.5%, and 34.2%, respectively, with most cases falling into the moderate level. Approximately 14% reported moderate and higher levels of depressive symptoms, 25.4% reported moderate and higher anxiety symptoms, and 20.2% reported moderate to severe levels of stress symptoms. Extremely severe levels were reported in the anxiety subscale only (Figure 1).

The prevalence of the symptoms of at least one subscale among the study cohort was 44.7% (n = 51), while the prevalence of all three was 16.7% (n = 19). Overall, the prevalence of two or more subscales among participants was higher than the prevalence of one condition only (Table 1). A statistically significant correlation between the subscales was found, indicating a highly significant association between them (*p* < 0.001) (Table 2). Furthermore, there was a positive, moderately strong, and statistically significant correlation among the subscales in the study cohort (*p* < 0.001) according to Spearman’s rank-order correlation test (Table 2) [22].

### 3.3. Descriptive Analysis of DASS Scores and Demographic Variables

Table 3 presents the mean (±SD) and median DASS-21 subscale scores across demographic variables, while Supplementary Tables S1–S3 summarize the distribution of participants across DASS severity categories. Overall, male and female participants exhibited comparable mean and median scores across subscales, with the exception of stress, where female participants reported higher mean scores (10.66 ± 7.02) than males (8.00 ± 7.74). Unmarried participants demonstrated higher mean stress (10.72 ± 6.68 vs. 8.97 ± 7.67) and depression scores (8.24 ± 5.20 vs. 7.57 ± 6.43) than married participants, whereas married individuals scored higher on anxiety (5.53 ± 6.05 vs. 4.00 ± 3.90).

Regarding academic rank, junior faculty (demonstrators and lecturers), followed by non-faculty participants exhibited higher mean and median DASS-21 scores across all three subscales. Mean scores decreased through the rank of associate professor, before showing a slight increase among professors (Table 3). Notably, the professor and non-faculty groups were the only groups to report extremely severe anxiety levels (Table S2). A breakdown by years of experience showed that overall DASS scores tended to be higher and more prevalent among junior dentists with less than five years of experience, then continued to decrease with more experience (except for the 11–15-year group), and was the lowest and least prevalent in the group with >20 years of experience (Table 3). Across dental specialties, general dental practitioners (n = 4) reported the highest mean scores across all three subscales (stress: 17.00 ± 10.65; anxiety: 14.00 ± 13.66; depression: 12.50 ± 8.85), followed by members of the oral medicine and diagnostic sciences, oral maxillofacial surgery, and prosthodontics specialties. In contrast, the lowest mean scores were observed in pediatric (stress: 3.20 ± 3.91; anxiety: 1.60 ± 2.07; depression: 3.80 ± 4.37) and orthodontics specialties.

### 3.4. Correlation of Subscales and Study Variables

A multiple linear regression model examined the correlation between study variables and each subscale. Categorical predictors were collapsed to reduce dummy variables. Satisfaction was collapsed into one variable. Subspecialty was not included in the model due to the large number of subgroups and a small number of cases within some of them. Bivariate screening was conducted using Mann–Whitney U tests, retaining variables with a *p*-value < 0.20. The demographic variable “marital status” returned a *p*-value > 0.20 and was excluded from the model. Additionally, “years of experience” was anticipated to demonstrate collinearity with “rank”, and only rank was included in the model, thereby adhering to the seven predictors, as calculated in the sample size. Gender, administrative roles, private practice, if first choice was academia, satisfaction, and rank were included in the regression model. Table 4 presents the regression findings.

#### 3.4.1. Depression and Anxiety

The regression equation (*R*^2^ = 0.114, *F*_(7,106)_ = 1.939, *p* = 0.070) was not statistically significant for the depression subscale, nor was the regression equation (*R*^2^ = 0.110, *F*_(7,106)_ = 1.878, *p* = 0.080) for the anxiety subscale statistically significant.

#### 3.4.2. Stress

A significant regression equation was found (*R*^2^ = 0.178, *F*_(7,106)_ = 3.279, *p* = 0.003). Three variables were significantly correlated with stress scores: satisfaction, gender, and choosing to work in academia as the first career choice (Table 4). Elevated stress scores were exhibited in female compared to male participants (b = 2.99, β = 0.201, *p* = 0.032) and in participants who were not satisfied compared to satisfied participants (b = 4.69, β = 0.265, *p* = 0.004), while stress scores were significantly lower in participants for whom working in academia was their first career choice (b = −2.70, β = −0.317, *p* = 0.030).

### 3.5. Methods of Stress Reduction

**Figure 2:**
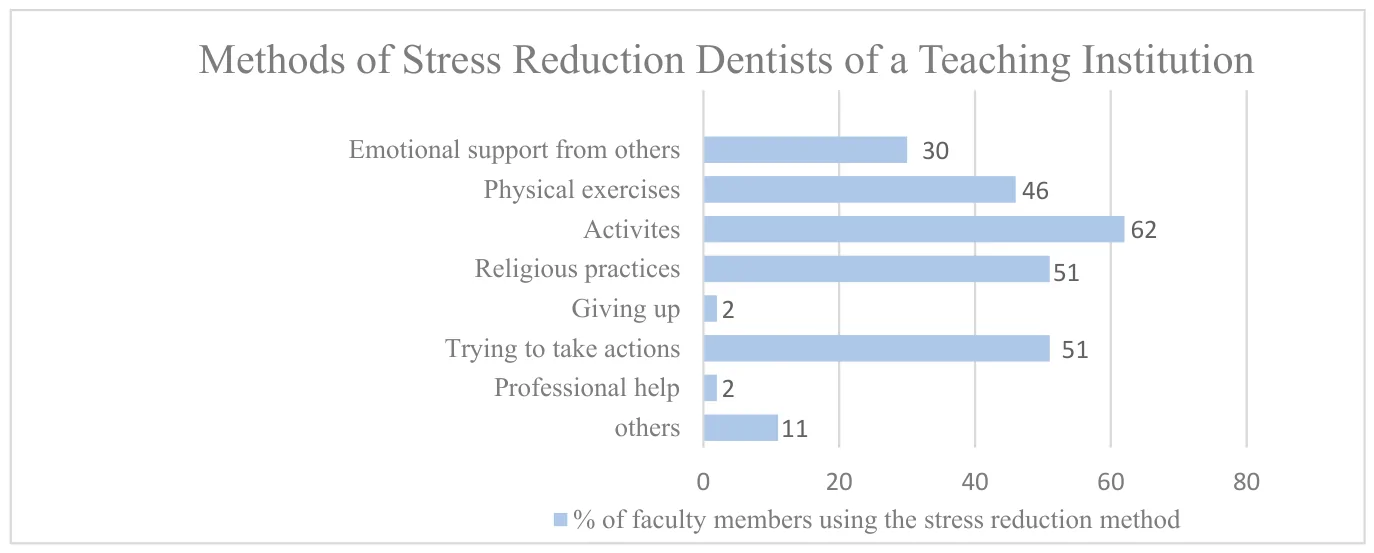
Bar chart illustrating the most frequently reported stress reduction methods among participants (N = 114). Percentages reflect the proportion of participants who selected each method; categories are not mutually exclusive, and percentages will not sum to 100%.

Figure 2 presents methods of stress reduction, as reported by participants: actively finding solutions to situations, engaging in various activities, and receiving support from others were the most common methods selected by participants. Bars represent the percentage of total respondents who selected each strategy. Percentages do not sum to 100% because participants could select multiple response options.

## 4. Discussion

This study provides the first explorative data on the prevalence of depressive, anxiety, and stress symptoms among dentists in academia in the Arab region (KSU), utilizing the validated DASS-21 scale, and it explores variables that may correlate with them. The findings suggest that dentists in academia experience considerable levels of depressive, anxiety, and stress symptoms, mostly at moderate levels.

The observed prevalence of depressive, anxiety, and stress symptoms in this sample reflects a significant mental health concern, which supports the dental and academic literature. The prevalence rates are consistent with the 18–20% depression range in meta-analyses of dentists and academic physicians, and comparable to the elevated anxiety and stress levels (30–40%) seen in regional healthcare cohorts [4,11]. Studies of dental educators in Europe and in Pakistan also support these elevated rates, though reported variations often stem from methodological differences in assessment tools [23,24]. However, using the same DASS-21 assessment tool, the prevalence of stress symptoms in this study (34.2%) exceeds earlier reports of 22–24% among general dentists [25], suggesting that unlike purely clinical dental practitioners, dental academics balance clinical pressures with inherent academic stressors, including heavy teaching and research loads, administrative demands, and the burden to continue working outside of working hours [12,26,27]. Similar to medical faculty, they often report feeling unappreciated, in addition to a lack of sufficient time for scholarly activities. These challenges may be further compounded by institutional issues, such as salary inequities, inadequate resources, and navigating clinical education without formal pedagogical training. Therefore, this multifaceted nature of the dental academic role can be a source of cumulative psychological strain [15,16]. Finally, the timing of studies plays a role, as faculty wellness reports from the COVID-19 era consistently show higher DASS scores, burnout and loneliness than pre-pandemic studies [28,29].

It is important to note the DASS-21 is a self-reporting screening tool and is not intended for clinical diagnosis. It is valuable in being rapid, has high sensitivity and validity and can be used to accurately detect individuals with depressive or anxiety symptoms and distinguish between them, but it cannot substitute clinical examination by an appropriate healthcare professional for diagnosis of depressive and anxiety disorders, which is standard practice to be achieved through the Diagnostic and Statistical Manual of Mental Disorders [6,30]. Prior psychometric evaluations in Saudi healthcare academic populations demonstrate that the English DASS-21 demonstrated high internal consistency and reliability (Cronbach’s α > 0.8 across all three subscales), avoiding linguistic or semantic shifts that can occur during translation [31].

The strong, positive correlations found among depression, anxiety, and stress levels in the present study are consistent with findings of high comorbidity among these conditions in high-demand occupations [11,20,23]. This significant co-occurrence highlights the need for comprehensive mental health strategies rather than interventions targeting a single condition.

The work-related and demographic factors that were found to be significantly correlated with stress scores provide valuable insights. Firstly, female participants reported significantly higher stress scores than male participants. Similarly, in a cross-sectional study in Saudi Arabia, female dental practitioners also scored higher in depression, anxiety and stress [28]. This pattern can be frequently observed globally among dentists, as well as dental and non-dental faculty [4,11,12,32]. Nesbitt et al. investigated the work environment for dental faculty and reported that females perceived their work environment to be more stressful, less supportive and less positive than their male colleagues. Female dentists also suffer more musculoskeletal problems, which, in turn, are associated with psychological problems [1,5].

Participants’ satisfaction with their relationships with students, colleagues, and the college overall was significantly correlated with symptoms of stress [33], as dissatisfied participants scored nearly 4.7 points higher on the Stress Scale. Indeed, Froeschele and Sinkford identified work relationships with colleagues and interactions with students, followed by administration and supporting staff, as the most positive workplace aspects for dental faculty [16]. On the contrary, administrative work, followed by clinical duties, was the most stressful, while most joy came from teaching [34]. Other major sources of dissatisfaction and stress are heavy workloads, time constraints, performing different roles, financial limitations, lack of institutional support, and a poor work–life balance [17,23,27,33]. In general, faculty are drawn to academia by a desire to teach, the appeal of an intellectual and scientific environment, and the influence of mentors [35]; therefore, the job satisfaction of staff should be addressed by institutions as it may protect mental health against job stress and burnout [36,37]. In this study, satisfaction with relationships was directly assessed on a Likert scale. It did not specifically measure job satisfaction or analyze the factors associated with it. It is suggested that job satisfaction should be measured using specialized tools in the future.

Similarly, participants for whom working in an academic institution was their first career choice reported significantly lower symptoms of stress. This aligns with the existing literature, which shows that physicians affiliated with academic institutions generally report better mental health than those who are not [37]. Research also demonstrates a strong inverse relationship between career fit, which is the alignment between professional identity and daily activities and the risk of burnout [15]. For example, conflict between self-identifying as an educator, a clinician, or an administrator can undermine job satisfaction [13,38]. Consequently, individuals who are more engaged in aspects of their career that provide a strong sense of purpose and that they find personally meaningful can be significantly less distressed [15,36,38].

The rank of participants was not significantly correlated with stress levels; this indicates that stress levels were not significantly different among junior and senior ranks. The literature has explored the nature of stressors across academic careers. Junior participants may face intense initial career stress as they build clinical, teaching, and research portfolios simultaneously, which is also observed across medicine and academia in general [15,17,24], while senior participants may face high-stakes pressure for tenure, scholarship output, and leadership [26]. These findings suggest that while the specific sources of stress vary over time, significant psychological distress could remain an occupational risk at every stage of the career in academia [26].

Descriptive observations by specialty highlighted differing score trends among participants. Studies suggest that dentists with specialized or advanced professional degrees often report greater satisfaction with their psychosocial work environment, including higher self-confidence and control over their work, which contributes to lower stress and anxiety compared to general practitioners [3,39]. However, these findings should be observed cautiously due to small sample sizes in some specialties; they also cannot be extrapolated to the general population as they were not tested for statistical significance.

Interestingly, none of the investigated variables were significantly associated with the depression and anxiety subscales despite their comparable prevalence and levels to stress. Although DASS-21 is highly sensitive to differences between the three subscales, this finding suggests that the variables included in the current regression model may not capture the primary predictors of depression and anxiety among dentists in academia. The non-significance of the models and various factors suggests that, in this specific cohort, distress may be predominantly an environmental phenomenon rather than an individual vulnerability, likely reflecting a high degree of cohort homogeneity regarding exposure to academic stressors [11]. Future research is suggested to explore structural and institutional metrics over demographic descriptors when modeling faculty well-being.

A critical implication of our findings is the considerable discrepancy between the high prevalence of psychological distress and the low proportion of participants receiving psychological management, although 44.7% of the sample reported psychological symptoms. Healthcare professionals have been known to be reluctant to receive support [39], which often stems from professional stigma, a culture of self-reliance, and fear of career repercussions [12,24,26].

The high prevalence of psychological distress among dentists in academia poses a critical challenge to institutional function and educational mission. The consequences extend beyond the individual, as impaired wellness is linked to less effective performance and an increased risk of clinical errors [8,26]. Within the academic environment, this burden directly impacts the quality of education, as faculty burnout is associated with reduced academic productivity and diminished teaching quality [23]. Furthermore, stressed educators may inadvertently model unhealthy habits for their students, thereby perpetuating a cycle of distress [23]. This is critical, given that educators are expected to support the prevalent mental health issues among dental students [31], a responsibility they may lack the emotional capacity to fulfill if their own well-being is compromised [40]. Given that educator well-being impacts student outcomes and clinical decision making, institutional strategies could explore barriers, reducing stigma and facilitating accessible, confidential support.

Addressing distress requires a strategic approach, starting with awareness of psychological well-being among staff, and early recognition and management of stress and timely intervention through structured mental health monitoring and wellness promotion [41,42]. Institutionally, a supportive work environment could be established, particularly for vulnerable groups such as female members [17,24]. Furthermore, it has been suggested the mitigating occupational stressors may benefit from balanced workload management, sufficient secretarial and research support, recognition, and improved administrative processes and salaries [16,33,35]. Ultimately, supporting the well-being of dentists in academia is essential to maintain clinical performance, teaching quality, and institutional resilience [8,26].

### Limitations and Future Directions

The present study has several notable strengths. The high response rate provides considerable representation, thereby minimizing non-response bias. The well-validated and psychometrically sound study tool (supported by the high internal consistency calculated in this study) ensures reliable measurement. Most importantly, the research addresses a critical need by providing quantitative data on a previously understudied, high-risk professional group within the region. However, this study is limited by its cross-sectional design, which restricts the interpretation of identified factors to correlations rather than causation. A second limitation stems from the self-reported measures of psychological distress, which may introduce social desirability bias and lead to an underestimation of the true prevalence, particularly when minimizing symptoms is professionally and culturally common. The exclusion of participants already receiving psychological management may add to this underestimation. Key psychosocial variables such as perceived workload, autonomy, and social support were beyond the scope of this study and should be incorporated into future investigations. Additionally, the single-institution nature restricts the generalizability of these findings. Finally, although the dataset reflects conditions in 2015, the findings remain highly relevant, as stressors within academic dentistry are largely unchanged, and the literature consistently reports rising levels of stress and burnout, particularly post-COVID-19, which led to an increase in psychological disturbances worldwide [26,29], suggesting the prevalence reported here may serve as a valuable baseline. Future longitudinal, multi-institutional studies utilizing objective measures alongside self-report tools are necessary to overcome these limitations.

## 5. Conclusions

Depressive, anxiety, and stress symptoms are prevalent among dentists at King Saud University, with female dentists, dissatisfied members and those who did not choose to work in academic institutions as their first career choice exhibiting particular vulnerability. As the first exploratory study in this population, these findings establish baseline evidence for future research. Institutions should aim to explore and enhance faculty well-being and maintain high standards in dental education and patient care.

## Figures and Tables

**Table 1:** Demographics of study participants in an academic dental institute and distribution of DAS subscale prevalence according to demographic variables (n = 114).

Prevalence of DAS Subscales			Total	Demographic Variable	
2 or 3 DAS Subscales	1 DAS	Normal	n (%)		
	Subscale	(No DAS)			
14 (25%)	7 (12.5%)	35 (62.5%)	56 (49.1%)	Male	**Gender**
19 (32.7%)	11 (19%)	28 (48.3%)	58 (50.9%)	Female	
6 (30%)	5 (25%)	9 (45%)	20 (17.5%)	Single	**Marital status**
26 (29.2%)	13 (14.6%)	50 (56.2%)	89 (78.1%)	Married	
1 (50%)	0	1 (50%)	2 (1.8%)	Widowed	
0	0	1 (100%)	1 (0.9%)	Divorced	
0	0	2 (100%)	2 (1.8%)	Missing	
6 (27.3%)	7 (31.8%)	9 (40.9%)	22 (19.3%)	Demonstrator	**Rank**
7 (25%)	7 (25%)	14 (50%)	28 (24.6%)	Lecturer	
4 (21.1%)	2 (10.5%)	13 (68.4%)	19 (16.7%)	Assistant Professor	
4 (22.2%)	2 (11.1%)	12 (66.7%)	18 (15.8%)	Associate Professor	
5 (41.7%)	0	7 (58.3%)	12 (10.5%)	Professor	
7 (46.7%)	0	8 (53.3%)	15 (13.2%)	Non-faculty	
10 (29.4%)	9 (26.5%)	15 (44.1%)	34 (29.8%)	<5	**Years of experience**
10 (33.3%)	6 (20%)	14 (46.7%)	30 (26.3%)	6–10	
3 (27.3%)	2 (18.2%)	6 (54.5%)	11 (9.6%)	11–15	
6 (33.3%)	0	12 (66.7%)	18 (15.8%)	16–20	
4 (19%)	1 (4.8%)	16 (76.2%)	21 (18.4%)	>20	
3 (75%)	0	1 (25%)	4 (3.5%)	General practitioner	**Specialty**
4 (22.2%)	3 (16.7%)	11 (61.1%)	18 (15.8%)	Operative	
2 (22.2%)	1 (11.1%)	6 (66.7%)	9 (7.9%)	Endodontics	
0	1 (10%)	9 (90%)	10 (8.8%)	Pediatric dentistry	
4 (33.3%)	1 (8.3%)	7 (58.3%)	12 (10.5%)	Periodontics	
9 (36%)	3 (12%)	13 (52%)	25 (21.9%)	Prosthodontics	
4 (50%)	0	4 (50%)	8 (7%)	Oral maxillofacial surgery	
4 (30.8%)	5 (38.5%)	4 (30.8%)	13 (11.4%)	Oral medicine and diagnostic sciences	
2 (28.6%)	1 (14.3%)	4 (57.1%)	7 (6.1%)	Orthodontics	
1 (12.5%)	3 (37.5%)	4 (50%)	8 (7%)	Dental public health and community	
24 (27.6%)	11 (12.6%)	52 (59.8%)	87 (76.3%)	Yes	**Academia was first career choice**
9 (34.6%)	7 (26.9%)	10 (38.5%)	26 (22.8%)	No	
10 (27.8%)	6 (16.7)	20 (55.5%)	36 (31.6%)	Yes	**Working also in private practice**
23 (29.5%)	12 (15.4%)	43 (55.1%)	78 (68.4%)	No	
3 (18.8%)	3 (18.8%)	10 (62.5%)	16 (14%)	0	**Number of administrative roles**
12 (30.8%)	8 (20.5%)	19 (48.7%)	39 (34.2%)	1–2	
15 (34.1%)	6 (13.6%)	23 (52.3%)	44 (38.6%)	3–4	
3 (20%)	1 (6.7%)	11 (73.3%)	15 (13.1%)	More than 4	
27 (27.3%)	15 (15.2%)	57 (57.6%)	99 (86.8%)	Satisfied	**Satisfaction with colleagues’ relationships**
3 (42.9%)	2 (28.6%)	2 (28.6%)	7 (6.1%)	Dissatisfied	
3 (37.5%)	1 (12.5%)	4 (50%)	8 (7.0%)	Neither	
29 (27.6%)	18 (17.1%)	58 (55.2%)	105 (92.1%)	Satisfied	**Satisfaction with students’ relationships**
1 (50%)	0	1 (50%)	2 (1.8%)	Dissatisfied	
3 (42.9%)	0	4 (57.1%)	7 (6.1%)	Neither	
28 (27.5%	16 (15.7%)	58 (56.9%)	102 (89.5%)	Satisfied	**Satisfaction with college overall**
3 (42.9%)	1 (14.3%)	3 (42.9%)	7 (6.1%)	Dissatisfied	
2 (40%)	1 (20%)	2 (40%)	5 (4.4%)	Neither	

**Table 2:** Prevalence of abnormal levels of DAS subscales, co-occurrence, and their correlations (n = 114).

Subscales		Depression n (%)		*p*-Value *	*p*-Value † (*r*_s_)	Anxiety n (%)		*p*-Value *	*p*-Value † (*r*_s_)	Stress n (%)		*p*-Value *	*p*-Value † (*r*_s_)
		Normal	Abnormal			Normal	Abnormal			Normal	Abnormal		
**Depression**	**Normal**	-	-	-		69 (89.6)	20 (54.1)	<0.001 *	**<0.001 †** (0.402)	72 (96.0)	17 (43.6)	<0.001 *	**<0.001 †** (0.601)
	**Abnormal**	-	-			8 (10.4)	**17 (45.9)**			3 (4.0)	**22 (56.4)**		
**Anxiety**	**Normal**	69 (77.5)	8 (32.0)	<0.001 *	**<0.001 †** (0.402)	-	-	-		65 (86.7)	12 (30.8)	<0.001 *	**<0.001 †** (0.566)
	**Abnormal**	20 (22.5)	**17 (68.0)**			-	-			10 (13.3)	**27 (69.2)**		
**Stress**	**Normal**	72 (80.9)	3 (12.0)	<0.001 *	**<0.001 †** (0.601)	65 (84.4)	10 (27.0)	<0.001*	**<0.001 †** (0.566)	-	-	-	
	**Abnormal**	17 (19.1)	**22 (88.0)**			12 (15.6)	**27 (73.0)**			-	-		

* Pearson’s Chi-square test, *p* < 0.05 is statistically significant. † Spearman’s rank-order correlation (***r*_s_**), *p* < 0.05 is statistically significant.

**Table 3:** DASS scores presented by mean, standard deviation, and median values for each demographic variable in the study population (n = 114).

Stress		Anxiety		Depression		n	Demographic Variable	
Median	Mean ± (SD)	Median	Mean ± (SD)	Median	Mean ± (SD)			
6	8.00 ± (7.74)	3	5.39 ± (6.51)	6	7.39 ± (6.43)	56	Male	**Gender**
10	10.66 ± (7.02)	5	5.00 ± (4.77)	6	8.03 ± (5.95)	58	Female	
8	8.97 ± (7.67)	4	5.53 ± (6.05)	6	7.57 ± (6.43)	89	Married	**Marital status**
10	10.72 ± (6.68)	2	4 ± (3.9)	6	8.24 ± (5.2)	25	Unmarried	
10	11.45 ± (7.54)	6	5.64 ± (4.77)	8	9.55 ± (7.38)	22	Demonstrator	**Rank**
10	10.50 ± (7.05)	5	4.50 ± (4.24)	6	7.64 ± (4.93)	28	Lecturer	
6	7.68 ± (6.84)	2	4.32 ± (4.91)	6	6.74 ± (5.51)	19	Assistant Professor	
5	7.22 ± (6.76)	2	4.22 ± (4.94)	4	5.44 ± (5.31)	18	Associate Professor	
6	7.50 ± (7.96)	1	6.00 ± (7.77)	5	7.67 ± (7.18)	12	Professor	
6	10.27 ± (9.00)	4	7.47 ± (8.53)	6	9.20 ± (7.04)	15	Non-faculty	
10	10.82 ± (7.69)	6	6.35 ± (6.58)	8	9.12 ± (6.74)	34	<5	**Years of experience**
10	10.73 ± (7.02)	4	4.47 ± (3.51)	8	8.53 ± (5.63)	30	6–10	
8	11.45 ± (7.75)	6	6.18 ± (6.16)	6	8.18 ± (6.35)	11	11–15	
4	8.33 ± (8.04)	1	5.11 ± (7.27)	6	6.22 ± (5.98)	18	16–20	
2	4.76 ± (5.42)	2	3.90 ± (4.79)	4	5.33 ± (5.60)	21	>20	
17	17.00 ± (10.65)	12	14.00 ± (13.66)	12	12.50 ± (8.85)	4	General practitioner	**Specialty**
9	9.33 ± (7.03)	2	3.11 ± (3.16)	6	6.56 ± (3.87)	18	Operative	
6	7.78 ± (7.38)	2	3.33 ± (4.12)	4	7.11 ± (8.07)	9	Endodontics	
2	3.20 ± (3.91)	1	1.60 ± (2.07)	4	3.80 ± (4.37)	10	Pediatric dentistry	
8	9.00 ± (7.36)	6	5.67 ± (5.03)	6	7.00 ± (5.62)	12	Periodontics	
10	9.76 ± (8.15)	6	6.08 ± (5.64)	8	8.72 ± (6.40)	25	Prosthodontics	
9	10.75 ± (7.78)	7	7.00 ± (5.35)	8	8.50 ± (6.74)	8	Oral maxillofacial surgery	
14	12.92 ± (6.36)	6	8.15 ± (5.97)	8	10.31 ± (5.41)	13	Oral medicine and diagnostic sciences	
8	8.29 ± (8.04)	0	2.00 ± (3.06)	4	5.14 ± (5.87)	7	Orthodontics	
6	8.00 ± (5.66)	4	4.75 ± (5.55)	6	8.75 ± (8.14)	8	Dental public health and community	
8	8.83 ± (7.49)	2	4.76 ± (5.74)	6	7.36 ± (6.31)	87	Yes	**Academia was first career choice**
12	11.38 ± (7.22)	6	6.85 ± (5.22)	8	9.08 ± (5.66)	26	No	
8	10.00 ± (8.49)	2	5.43 ± (6.05)	6	7.54 ± (6.65)	36	Yes	**Working also in private practice**
8	9.15 ± (7.01)	4	5.15 ± (5.53)	6	7.85 ± (6.01)	78	No	
3	5.88 ± (6.47)	1	3.38 ± (4.77)	3	4.50 ± (5.03)	16	0	**Number of administrative roles**
10	10.26 ± (7.90)	6	6.00 ± (6.19)	8	8.97 ± (6.77)	39	1–2	
8	10.27 ± (7.29)	4	5.73 ± (5.78)	6	8.55 ± (6.29)	44	3–4	
6	8.00 ± (7.25)	2	3.47 ± (4.31)	6	5.47 ± (3.25)	15	More than 4	
8	8.99 ± (7.33)	2	4.91 ± (5.68)	6	7.25 ± (5.97)	99	Satisfied	**Satisfaction with colleagues’ relationships**
14	14.57 ± (8.77)	8	7.71 ± (6.58)	12	13.43 ± (6.80)	7	Dissatisfied	
10	9.25 ± (7.40)	6	6.50 ± (4.50)	7	8.50 ± (6.30)	8	Neither	
8	9.03 ± (7.38)	4	5.18 ± (5.71)	6	7.50 ± (6.00)	105	Satisfied	**Satisfaction with students’ relationships**
14	11.71 ± (7.34)	6	6.57 ± (5.62)	6	9.71 ± (8.75)	7	Neither	
18	18.00 ± (11.31)	1	1.00 ± (1.41)	12	12.00 ± (5.66)	2	Dissatisfied	
8	9.06 ± (7.32)	4	4.84 ± (5.12)	6	7.49 ± (6.05)	102	Satisfied	**Satisfaction with college overall**
10	12.29 ± (9.69)	8	8.86 ± (10.88)	8	8.57 ± (7.18)	7	Dissatisfied	
14	11.20 ± (7.95)	6	7.20 ± (6.10)	8	11.20 ± (7.56)	5	Neither	

**Table 4:** Multiple linear regression model for prediction of depression, anxiety, and stress among dentists in a teaching institute (n = 114).

Predictor		Subscale	Unstandardized Coefficients		Standardized Coefficients	95.0% Confidence Interval for B		Collinearity Statistics		t	*p*-Value
			b	SE	β	Lower Bound	Upper Bound	Tolerance	VIF		
**Constant**		D	4.336	1.760		0.874	7.825			2.464	0.015
		A	4.420	1.619		1.210	7.631			2.730	0.007
		S	5.102	2.052		1.034	9.170			2.487	0.014
**Gender**	Male	Reference	-	-	-					-	-
	Female	D	0.735	1.181	0.060	−1.607	3.077	0.905	1.105	0.622	0.535
		A	0.002	1.087	0.000	−2.153	2.157			0.002	0.999
		S	2.989	1.377	0.201	0.258	5.719			2.170	0.032 *
**Admin Roles**	No	Reference									
	Yes	D	3.061	1.825	0.173	−0.557	6.679	0.786	1.273	1.678	0.096
		A	1.847	1.679	0.114	−1.482	5.176			1.100	0.274
		S	2.638	2.128	0.123	−1.580	6.856			1.240	0.218
**Working in academia first career choice**	No	Reference									
	Yes	D	−1.172	1.054	−0.167	−3.261	0.917	0.372	2.690	−1.112	0.269
		A	−1.808	0.970	−0.280	−3.731	0.114			−1.865	0.065
		S	−2.700	1.229	−0.317	−5.136	−0.264			−2.197	0.030 *
**Working in private practice**	No	Reference									
	Yes	D	0.584	0.986	0.089	−1.371	2.54	0.373	2.678	0.592	0.555
		A	0.885	0.908	0.146	−0.915	2.684			0.975	0.332
		S	1.939	1.150	0.243	−0.341	4.220			1.686	0.095
**Satisfaction**	Satisfied	Reference									
	Other	D	3.220	1.354	0.220	0.535	5.905	0.978	1.023	2.377	0.019 *
		A	2.440	1.246	0.181	−0.030	4.911			1.959	**0.053**
		S	4.693	1.579	0.265	1.563	7.824			2.972	0.004 *
**RANK**	Senior	Reference									
	Junior	D	0.328	1.487	0.026	−2.620	3.276	0.597	1.674	0.221	0.826
		A	−0.783	1.368	−0.068	−3.496	1.930			−0.572	0.568
		S	1.072	1.734	0.070	−2.365	4.510			0.618	0.538
	Non-faculty	D	1.570	2.106	0.086	−2.607	5.746	0.623	1.606	0.745	0.458
		A	2.144	1.938	0.128	−1.698	5.987			1.106	0.271
		S	2.023	2.456	0.092	−2.846	6.892			0.824	0.412

Note: * Significant predictor (*p* < 0.05). D = depression, A = anxiety, S = stress, Senior = Associate and full professors, Junior = Demonstrators, lecturers and assistant professors.

## Data Availability

All data have been presented as aggregated tables in the manuscript and Supplementary File, due to the nature of the confidentiality agreement with the study participants.

## Supplementary Materials

The following supporting information can be downloaded at: https://www.mdpi.com/article/10.3390/healthcare14182908/s1, Table S1. Distribution (n, %) of depression subscale of DASS-21 within participant demographics (n = 114); Table S2. Distribution (n, %) of anxiety subscale of DASS-21 within participant demographics (n = 114); Table S3. Distribution (n, %) of stress subscale of DASS-21 within participant demographics (n = 114).
