# Supplementary material for: Prevalence of Depressive, Anxiety, and Stress Symptoms Among Dentists at an Academic Institution: A Cross-Sectional Study
Source: Healthcare (Basel). 2026 Sep 8;14(18):2908. doi: 10.3390/healthcare14182908 (PMC13606251; doi:10.3390/healthcare14182908)
Supplement: Supplementary file 1 [file healthcare-14-02908-s001.zip › healthcare-4491913-supplementary.pdf]

Supplementary File

Table S1. Distribution (n, %) of depression subscale of DASS-21 within participant demographics (n=114)

Demographic variable		Depression Subscale Levels			
		Normal	Mild	Moderate	Severe
**Gender**	Male	39 (69.6%)	6 (10.7%)	8 (14.3%)	3 (5.4%)
	Female	35 (60.3%)	10 (17.2%)	11 (19.0%)	2 (3.4%)
**Marital status**	Unmarried	17 (68.0%)	3 (12.0%)	5 (20.0%)	0 (0.0%)
	Married	57 (64.0%)	13 (14.6%)	14 (15.7%)	5 (5.6%)
**Rank**	Demonstrator	14 (63.6%)	2 (9.1%)	3 (13.6%)	3 (13.6%)
	Lecturer	17 (60.7%)	6 (21.4%)	5 (17.9%)	0 (0.0%)
	Assistant Professor	14 (73.7%)	3 (15.8%)	1 (5.3%)	1 (5.3%)
	Associate Professor	13 (72.2%)	2 (11.1%)	3 (16.7%)	0 (0.0%)
	Professor	7 (58.3%)	1 (6.7%2	3 (25.0%)	0 (0.0%)
	Non-faculty	9 (60.0%)	1 (6.7%)	4 (26.7%)	1 (6.7%)
**Years of experience**	< 5	21 (61.8%)	5 (14.7%)	5 (14.7%)	3 (8.8%)
	6-10	15 (50.0%)	8 (26.7%)	6 (20.0%)	1 (3.3%)
	11-15	8 (72.7%)	0 (0.0%)	2 (18.2%)	1 (9.1%)
	16-20	14 (77.8%)	1 (5.6%)	3 (16.7%)	0 (0.0%)
	> 20	16 (76.2%)	2 (9.5%)	3 (14.3%)	0 (0.0%)
**Specialty**	General practitioner	2 (50.0%)	0 (0.0%)	1 (25.0%)	1 (25.0%)
	Operative	13 (72.2%)	4 (22.2%)	1 (5.6%)	0 (0.0%)
	Endodontics	6 (66.7%)	0 (0.0%)	2 (22.2%)	1 (11.1%)
	Pediatric dentistry	9 (90.0%)	0 (0.0%)	1 (10.0%)	0 (0.0%)
	Periodontics	7 (58.3%)	3 (25.0%)	2 (16.7%)	0 (0.0%)
	Prosthodontics	16 (64.0%)	4 (16.0%)	3 (12.0%)	2 (8.0%)
	Oral maxillofacial surgery	4 (50.0%)	2 (25.0%)	2 (25.0%)	0 (0.0%)
	Oral medicine and diagnostic sciences	7 (53.8%)	1 (7.7%)	5 (38.5%)	0 (0.0%)
	Orthodontics	5 (71.4%)	1 (14.3%)	1 (14.3%)	0 (0.0%)
	Dental public health and community	5 (62.5%)	1 (12.5%)	1 (12.5%)	1 (12.5%)
**Academia was first career choice**	Yes	58 (66.7%)	14 (16.1%)	10 (11.5%)	5 (5.7%)
	No	15 (57.7%)	2 (7.7%)	9 (34.6%)	0 (0.0%)
**Working also in private practice**	Yes	23 (65.7%)	4 (11.4%)	6 (17.1%)	2 (5.7%)
	No	50 (64.1%)	12 (15.4%)	13 (16.7%)	3 (3.8%)
**Number of administrative roles**	0	12 (75.0%)	3 (18.8%)	1 (6.3%)	0 (0.0%)
	1-2	23 (59.0%)	5 (12.8%)	8 (20.5%)	3 (7.7%)
	3-4	26 (59.1%)	6 (13.6%)	10 (22.7%)	2 (4.5%)
	More than 4	13 (86.7%)	2 (13.3%)	0 (0.0%)	0 (0.0%)
**Satisfaction with colleagues' relationships**	Satisfied	66 (66.7%)	13 (13.1%)	17 (17.2%)	3 (3.0%)
	Dissatisfied	5 (62.5%)	1 (12.5%)	2 (25.0%)	0 (0.0%)
	Neither	3 (42.9%)	2 (28.6%)	0 (0.0%)	2 (28.6%)
**Satisfaction with students' relationships**	Satisfied	69 (65.7%)	16 (15.2%)	16 (15.2%)	4 (3.8%)
	Dissatisfied	4 (57.1%)	0 (0.0%)	2 (28.6%)	1 (14.3%)
	Neither	1 (50.0%)	0 (0.0%)	1 (50.0%)	0 (0.0%)
**Satisfaction with college overall**	Satisfied	66 (64.7%)	15 (14.7%)	18 (17.6%)	3 (2.9%)
	Dissatisfied	5 (71.4%)	1 (14.3%)	0 (0.0%)	1 (14.3%)
	Neither	3 (60.0%)	0 (0.0%)	1 (20.0%)	1 (20.0%)

Table S2. Distribution (n, %) of anxiety subscale of DASS-21 within participant demographics (n=114)

Demographic variable		Anxiety Subscale Levels				
		Normal	Mild	Moderate	Severe	Extremely severe
**Gender**	Male	39 (69.6%)	7 (12.5%)	5 (8.9%)	3 (5.4%)	2 (3.6%)
	Female	41 (70.7%)	10 (17.2%)	4 (6.9%)	3 (5.2%)	0 (0.0%)
**Marital status**	Unmarried	19 (76.0%)	5 (20.0%)	1 (4.0%)	0 (0.0%)	0 (0.0%)
	Married	61 (68.5%)	12 (13.5%)	8 (9.0%)	6 (6.7%)	2 (2.2%)
**Rank**	Demonstrator	13 (59.1%)	7 (31.8%)	1 (4.5%)	1 (4.5%)	0
	Lecturer	22 (78.6%)	4 (14.3%)	1 (3.6%)	1 (3.6%)	0
	Assistant Professor	15 (78.9%)	2 (10.5%)	1 (5.3%)	1 (5.3%)	0
	Associate Professor	14 (77.8%)	2 (11.1%)	1 (5.6%)	1 (5.6%)	0
	Professor	7 (58.3%)	1 (8.3%)	2 (16.7%)	1 (8.3%)	1 (6.7%)
	Non-faculty	9 (60.0%)	1 (6.7%)	3 (20.0%)	1 (6.7%)	1 (6.7%)
**Years of experience**	< 5	20 (58.8%)	8 (23.5%)	4 (11.8%)	1 (2.9%)	1 (2.9%)
	6-10	24 (80.0%)	4 (13.3%)	2 (6.7%)	0 (0.0%)	0 (0.0%)
	11-15	7 (63.6%)	2 (18.2%)	0 (0.0%)	2 (18.2%)	0 (0.0%)
	16-20	12 (66.7%)	2 (11.1%)	1 (5.6%)	2 (11.1%)	1 (5.6%)
	> 20	17 (81.0%)	1 (4.8%)	2 (9.5%)	1 (4.8%)	0 (0.0%)
**Specialty**	General practitioner	1 (25.0%)	1 (25.0%)	0 (0.0%)	1 (25.0%)	1 (25.0%)
	Operative	16 (88.9%)	2 (11.1%)	0 (0.0%)	0 (0.0%)	0 (0.0%)
	Endodontics	7 (77.8%)	1 (11.1%)	1 (11.1%)	0 (0.0%)	0 (0.0%)
	Pediatric dentistry	10 (100.0%)	0 (0.0%)	0 (0.0%)	0 (0.0%)	0 (0.0%)
	Periodontics	9 (75.0%)	1 (8.3%)	1 (8.3%)	1 (8.3%)	0 (0.0%)
	Prosthodontics	14 (56.0%)	6 (24.0%)	3 (12.0%)	2 (8.0%)	0 (0.0%)
	Oral maxillofacial surgery	4 (50.0%)	1 (12.5%)	3 (37.5%)	0 (0.0%)	0 (0.0%)
	Oral medicine and diagnostic sciences	7 (53.8%)	3 (23.1%)	1 (7.7%)	1 (7.7%)	1 (7.7%)
	Orthodontics	6 (85.7%)	1 (14.3%)	0 (0.0%)	0 (0.0%)	0 (0.0%)
	Dental public health and community	6 (75.0%)	1 (12.5%)	0 (0.0%)	1 (12.5%)	0 (0.0%)
**Academia was first career choice**	Yes	65 (74.7%)	12 (13.8%)	4 (4.6%)	4 (4.6%)	2 (2.3%)
	No	14 (53.8%)	5 (19.2%)	5 (19.2%)	2 (7.7%)	0 (0.0%)
**Working also in private practice**	Yes	24 (68.6%)	5 (14.3%)	2 (5.7%)	3 (8.6%)	1 (2.9%)
	No	55 (70.5%)	12 (15.4%)	7 (9.0%)	3 (3.8%)	1 (1.3%)
**Number of administrative roles**	0	12 (75.0%)	3 (18.8%)	0 (0.0%)	1 (6.3%)	0 (0.0%)
	1-2	26 (66.7%)	6 (15.4%)	5 (12.8%)	1 (2.6%)	1 (2.6%)
	3-4	30 (68.2%)	6 (13.6%)	3 (6.8%)	4 (9.1%)	1 (2.3%)
	More than 4	12 (80.0%)	2 (13.3%)	1 (6.7%)	0 (0.0%)	0 (0.0%)
**Satisfaction with colleagues' relationships**	Satisfied	72 (72.7%)	15 (15.2%)	5 (5.1%)	5 (5.1%)	2 (2.0%)
	Dissatisfied	5 (62.5%)	1 (12.5%)	2 (25.0%)	0 (0.0%)	0 (0.0%)
	Neither	3 (42.9%)	1 (14.3%)	2 (28.6%)	1 (14.3%)	0 (0.0%)
**Satisfaction with students' relationships**	Satisfied	73 (69.5%)	17 (16.2%)	8 (7.6%)	5 (4.8%)	2 (1.9%)
	Dissatisfied	5 (71.4%)	0 (0.0%)	1 (14.3%)	1 (14.3%)	0 (0.0%)
	Neither	2 (100.0%)	0 (0.0%)	0 (0.0%)	0 (0.0%)	0 (0.0%)
**Satisfaction with college overall**	Satisfied	74 (72.5%)	13 (12.7%)	9 (8.8%)	5 (4.9%)	1 (1.0%)
	Dissatisfied	3 (42.9%)	3 (42.9%)	0 (0.0%)	0 (0.0%)	1 (14.3%)
	Neither	3 (60.0%)	1 (20.0%)	0 (0.0%)	1 (20.0%)	0 (0.0%)

Table S3. Distribution (n, %) of stress subscale of DASS-21 within participant demographics (n=114)

Demographic variable		Stress Subscale Levels			
		Normal	Mild	Moderate	Severe
**Gender**	Male	45 (80.4%)	6 (10.7%)	3 (5.4%)	2 (3.6%)
	Female	40 (69.0%)	12 (20.7%)	5 (8.6%)	1 (1.7%)
**Marital status**	Unmarried	18 (72.0%)	6 (24.0%)	0 (0.0%)	1 (4.0%)
	Married	67 (75.3%)	12 (13.5%)	8 (9.0%)	2 (2.2%)
**Rank**	Demonstrator	16 (72.7%)	4 (18.2%)	1 (4.5%)	1 (4.5%)
	Lecturer	21 (75.0%)	4 (14.3%)	2 (7.1%)	1 (3.6%)
	Assistant Professor	16 (84.2%)	1 (5.3%)	2 (10.5%)	0 (0.0%)
	Associate Professor	14 (77.8%)	3 (16.7%)	1 (5.6%)	0 (0.0%)
	Professor	9 (75.0%)	2 (16.7%)	1 (8.3%)	0 (0.0%)
	Non-faculty	9 (60.0%)	4 (26.7%)	1 (6.7%)	1 (6.7%)
**Years of experience**	< 5	26 (76.5%)	5 (14.7%)	1 (2.9%)	2 (5.9%)
	6-10	21 (70.0%)	5 (16.7%)	3 (10.0%)	1 (3.3%)
	11-15	7 (63.6%)	2 (18.2%)	2 (18.2%)	0 (0.0%)
	16-20	12 (66.7%)	4 (22.2%)	2 (11.1%)	0 (0.0%)
	> 20	19 (90.5%)	2 (9.5%)	0 (0.0%)	0 (0.0%)
**Specialty**	General practitioner	1 (25.0%)	2 (50.0%)	0 (0.0%)	1 (25.0%)
	Operative	14 (77.8%)	3 (16.7%)	0 (0.0%)	1 (5.6%)
	Endodontics	7 (77.8%)	1 (11.1%)	1 (11.1%)	0 (0.0%)
	Pediatric dentistry	10 (100.0%)	0 (0.0%)	0 (0.0%)	0 (0.0%)
	Periodontics	9 (75.0%)	2 (16.7%)	1 (8.3%)	0 (0.0%)
	Prosthodontics	18 (72.0%)	4 (16.0%)	2 (8.0%)	1 (4.0%)
	Oral maxillofacial surgery	5 (62.5%)	2 (25.0%)	1 (12.5%)	0 (0.0%)
	Oral medicine and diagnostic sciences	9 (69.2%)	2 (15.4%)	2 (15.4%)	0 (0.0%)
	Orthodontics	5 (71.4%)	1 (14.3%)	1 (14.3%)	0 (0.0%)
	Dental public health and community	7 (87.5%)	1 (12.5%)	0 (0.0%)	0 (0.0%)
**Academia was first career choice**	Yes	67 (77.0%)	12 (13.8%)	6 (6.9%)	2 (2.3%)
	No	17 (65.4%)	6 (23.1%)	2 (7.7%)	1 (3.8%)
**Working also in private practice**	Yes	26 (74.3%)	3 (8.6%)	4 (11.4%)	2 (5.7%)
	No	58 (74.4%)	15 (19.2%)	4 (5.1%)	1 (1.3%)
**Number of administrative roles**	0	14 (87.5%)	2 (12.5%)	0 (0.0%)	0 (0.0%)
	1-2	28 (71.8%)	7 (17.9%)	2 (5.1%)	2 (5.1%)
	3-4	32 (72.7%)	6 (13.6%)	5 (11.4%)	1 (2.3%)
	More than 4	11 (73.3%)	3 (20.0%)	1 (6.7%)	0 (0.0%)
**Satisfaction with colleagues' relationships**	Satisfied	75 (75.8%)	15 (15.2%)	7 (7.1%)	2 (2.0%)
	Dissatisfied	6 (75.0%)	1 (12.5%)	1 (12.5%)	0 (0.0%)
	Neither	4 (57.1%)	2 (28.6%)	0 (0.0%)	1 (14.3%)
**Satisfaction with students' relationships**	Satisfied	80 (76.2%)	16 (15.2%)	7 (6.7%)	2 (1.9%)
	Dissatisfied	4 (57.1%)	2 (28.6%)	1 (14.3%)	0 (0.0%)
	Neither	1 (50.0%)	0 (0.0%)	0 (0.0%)	1 (50.0%)
**Satisfaction with college overall**	Satisfied	78 (76.5%)	14 (13.7%)	8 (7.8%)	2 (2.0%)
	Dissatisfied	4 (57.1%)	2 (28.6%)	0 (0.0%)	1 (14.3%)
	Neither	3 (60.0%)	2 (40.0%)	0 (0.0%)	0 (0.0%)
